# Molecular-level protein semantic learning via structure-aware coarse-grained language modeling

**DOI:** 10.1093/bioinformatics/btaf654

**Published:** 2025-12-06

**Authors:** Jun Zhang, Xueer Weng, Tiantian Zhu, Yumeng Liu, Zexuan Zhu

**Affiliations:** School of Artificial Intelligence, Shenzhen University, Shenzhen, Guangdong 518060, China; National Engineering Laboratory for Big Data System Computing Technology, Shenzhen University, Shenzhen, Guangdong 518060, China; School of Artificial Intelligence, Shenzhen University, Shenzhen, Guangdong 518060, China; National Engineering Laboratory for Big Data System Computing Technology, Shenzhen University, Shenzhen, Guangdong 518060, China; School of Artificial Intelligence, Shenzhen University, Shenzhen, Guangdong 518060, China; National Engineering Laboratory for Big Data System Computing Technology, Shenzhen University, Shenzhen, Guangdong 518060, China; School of Artificial Intelligence, Shenzhen Technology University, Shenzhen, Guangdong 518118, China; School of Artificial Intelligence, Shenzhen University, Shenzhen, Guangdong 518060, China; National Engineering Laboratory for Big Data System Computing Technology, Shenzhen University, Shenzhen, Guangdong 518060, China

## Abstract

**Motivation:**

Protein language models (PLMs) have emerged as pivotal tools for protein representation, enabling significant advances in structure-function prediction and computational biology. However, current PLMs predominantly rely on fine-grained amino acid sequences as input, treating individual residues as tokens. While this approach facilitates semantic learning at the residue level, it struggles to capture molecular-level semantics, particularly for large proteins, where sequence truncation and inefficient local pattern extraction hinder holistic understanding. The spatial structure of a protein determines its function. Despite the critical role of protein function analysis, coarse-grained protein language frameworks that bridge sequence and structural semantics remain underdeveloped.

**Results:**

To fill this gap, we introduce a novel structure-aware coarse-grained protein language that discretizes proteins into local structural patterns derived from their secondary structures. By constructing a vocabulary of these patterns as “words,” we represent proteins as compact, structure-aware “sentences” significantly shorter than raw amino acid sequences. We benchmark the proposed coarse-grained language against three state-of-the-art fine-grained protein languages and a classical language modeling method in natural language processing, using two architectures: a lightweight Doc2Vec model and a Transformer-based BERT model, and evaluating performance across diverse downstream tasks, including function prediction, enzyme classification, and interaction identification. The proposed method achieves stable performance across three tasks, especially for long proteins. These results demonstrate that the proposed coarse-grained protein language preserves critical structural and functional semantics and improves molecular-level analysis, offering a promising direction for decoding higher-order biological insights.

**Availability and implementation:**

The data and source code of the proposed method are available at GitHub (https://github.com/bug-0x3f/coarse-grained-protein-language) and Zenodo (DOI: 10.5281/zenodo.17674298).

## 1 Introduction

Proteins are essential to life, driving virtually every biological process—from catalyzing metabolic reactions to enabling cellular communication. Deciphering their properties and functions advances our understanding of biology and holds transformative potential for applications such as disease therapy (Santos *et al.* 2017, Hanahan 2022), drug development (Scott *et al.* 2012), and engineered protein design (Arnold 2018).

A central challenge in computational protein science is representing these complex molecules in ways that capture their functional and structural nuances. Drawing inspiration from natural language processing (NLP), researchers have developed protein language models (PLMs) that translate amino acid sequences into numerical embeddings. Pioneering models like ESM (Meier *et al.* 2021, Rives *et al.* 2021, Lin *et al.* 2023), ProteinBERT (Brandes *et al.* 2022), and ProtTrans (Elnaggar *et al.* 2022) treat amino acids as discrete “words” and sequences as “sentences,” achieving breakthroughs in predicting protein structures, functions, and interactions.

However, protein function is intrinsically tied to its three-dimensional structure, which traditional sequence-based PLMs struggle to incorporate. Historically, this limitation arose from scarce structural data (Zhang *et al.* 2023). Recent advances in AI-driven tools like AlphaFold (Jumper *et al.* 2021) now provide reliable structural predictions, enabling models to integrate structural insights. Yet, unifying sequence and structural information into holistic protein representations remains an open challenge.

Recent progress in developing PLM has shifted focus toward optimizing the “language” used to represent proteins. For instance, SaProt (Su *et al.* 2023) converts structural data into discrete identifiers using FoldSeek (Van Kempen *et al.* 2024), merging them with sequence tokens to create structure-aware vocabularies. Similarly, ProSST (Li *et al.* 2024) quantizes structural features into tokens and uses specialized attention mechanisms to model residue-structure interactions. These efforts highlight the growing interest in structural integration while retaining a fine-grained tokenization approach, in which individual amino acids serve as tokens.

While residue-level modeling enables detailed analysis, it struggles to capture molecular-level semantics. The conserved functional domains, structurally stable regions, are critical to protein function (Eddy 2011, Finn *et al.* 2016). However, these domains, such as binding or catalytic regions (Ashburner *et al.* 2000, Orengo *et al.* 2003), are often poorly captured by fine-grained tokenization, limiting models’ ability to infer functionality (Senior *et al.* 2020). Furthermore, long protein sequences frequently exceed model input limits, forcing truncation and loss of global context. The small vocabulary size (20 amino acids) may also restrict the learning of higher-order functional patterns, as suggested by NLP scaling laws (Tao *et al.* 2024).

To address these limitations, we propose a Structure-aware, Coarse-Grained (SCG) protein language modeling framework. Instead of individual residues, we define “words” as local structural patterns derived from secondary structure segmentation. By clustering these patterns into a vocabulary via a vector quantization autoencoder, we represent proteins as concise, structure-aware “sentences” that retain biological meaning while drastically reducing the sequence length. We validate this approach by training language models on the SCG protein corpus and evaluating performance across three protein function prediction tasks: function prediction, enzyme classification, and interaction identification. The proposed framework achieves stable performance across three tasks, especially for long proteins.

The contributions of this study can be summarized as follows:

1. **Secondary structure-based segmentation**: We designed a method to divide proteins into biologically meaningful structural patterns, reducing the sequence length for efficient modeling;
2. **Structure-aware vocabulary construction**: We developed a vector quantization approach to map structural patterns to a fixed vocabulary, enabling structure-aware language modeling;
3. **Coarse-grained protein language**: We proposed the SCG protein language modeling framework. Comprehensive benchmarking demonstrates its superiority over fine-grained methods in function prediction tasks, offering a promising direction for decoding higher-order biological insights.

## 2 Materials and methods

**Figure 1.**
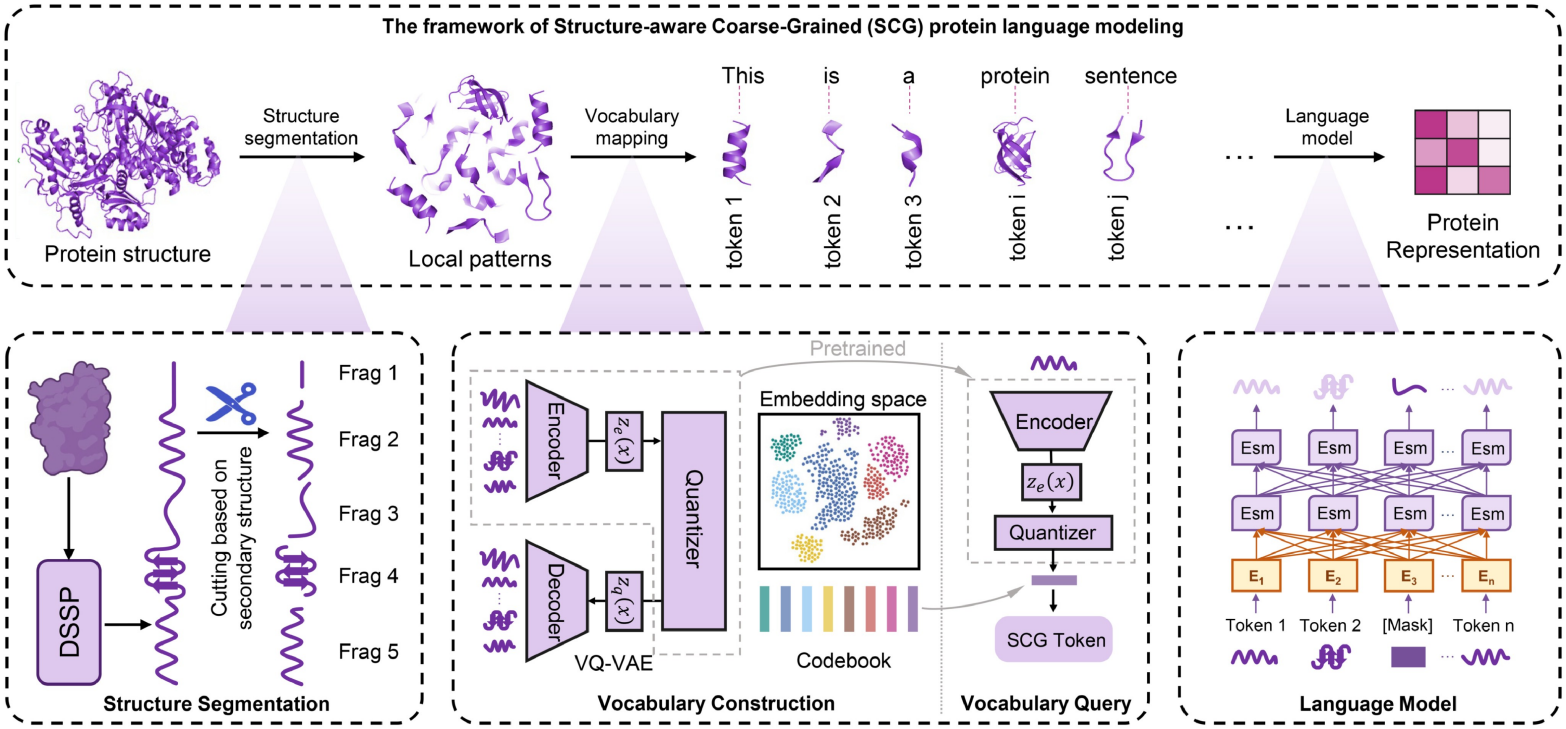
The framework of structure-based coarse-grained language modeling. This framework transforms protein into semantically enriched representations through three stages: (i) **Structure Segmentation**: Division of the protein structure into geometrically coherent fragments. (ii) **Vocabulary Query**: Conversion of fragments into discrete tokens via a pre-trained VQ-VAE, which maps structural information into a quantized embedding space through codebook-based compression. (iii) **Language Modeling**: Learning of hierarchical semantic patterns from the coarse-grained token sequences.

The proposed structure-aware coarse-grained protein language modeling framework is illustrated in Fig. 1, with the specific details outlined in the following sections.

### 2.1 Structure segmentation

For a protein structure, we utilize DSSP (Kabsch and Sander 1983) to extract the secondary structure profile, obtaining secondary structure information for each residue in the protein. The DSSP tool classifies protein secondary structures into eight types: helix (H), bridge (B), extended strand (E), 3-helix (G), 5-helix (I), hydrogen-bonded turn (T), bend (S), and coil or irregular loop (officially denoted as a space). Based on the secondary structure of the protein, we segment the protein structure by grouping consecutive amino acid residues within the sequence that share the same secondary structure type into a fragment. Each fragment with corresponding spatial conformation forms a local structural pattern.

### 2.2 Vocabulary construction

From the perspective of language design rationality, treating each secondary structure fragment obtained as a unique protein word is impractical, as it would result in an exponential explosion in vocabulary size. In natural language processing, an excessively large vocabulary hinders computational efficiency and increases the inference cost of the model (Sennrich *et al.* 2016, Lan *et al.* 2020). Moreover, some protein fragments exhibit substantial similarity, analogous to the homology observed among protein sequences. Therefore, it is essential to construct a non-redundant vocabulary with an appropriate size to facilitate protein language modeling.

The codebook mechanism in Vector Quantized Variational Autoencoder (VQ-VAE) (van den Oord *et al.* 2017) provides an implementation approach for this, which was initially used to encode images into discrete vectors, providing a new insight for image generation tasks and later inspiring various studies, including language modeling.

A VQ-VAE framework consists of three primary components: an encoder, a quantization model, and a decoder. During the encoding process, the input feature x is first mapped by the encoder into a continuous latent space $z_{e}$. The quantization process involves mapping each continuous latent vector $z_{e}$ to the nearest code vector in a predefined, learnable codebook $C={\left\{e_{i}\right\}}_{i=1}^{K}$, where *K* represents the number of codebook entries, and each $e_{i}\in R^{d}$ is a code vector that defines the vocabulary. Specifically, the quantized latent code $z_{q}$ is determined by finding the index *k* of the nearest code vector $e_{k}$ to $z_{e}$, which is computed as:


(1) $$k=\arg\underset{j}{\min}∥z_{e}-e_{j}{∥}_{2}$$

Thus, the quantized code $z_{q}$ corresponds to the index *k* of the selected code vector $e_{k}$ in the codebook. We treat his index *k* effectively as a “word” in the learned coarse-grained protein language vocabulary.

Since the quantization operation involves a discrete mapping, it is inherently non-differentiable. To enable end-to-end training, the straight-through estimator (STE) (Bengio *et al.* 2013) is employed to approximate gradients during backpropagation. The overall optimization objective of the VQ-VAE is to minimize the following loss function:


(2) $$L=L_{\text{recons}}+L_{\text{codebook}}+\beta L_{\text{commit}},$$

where each term contributes to different aspects of the model’s training.

The first term, $L_{\text{recons}}$, denotes the reconstruction loss, ensuring that the decoder accurately reconstructs the input from the quantized latent space. We use the Kullback-Leibler (KL) divergence as the reconstruction loss:


(3) $$L_{\text{recons}}=\text{KL}(p(x)∥q(\hat{x})),$$

where p(x) is the true distribution of the input data, and $q(\hat{x})$ represents the distribution of the reconstructed data. The KL divergence quantifies how closely the reconstructed distribution aligns with the original, driving the encoder and decoder to model the input data jointly and accurately.

The second term, $L_{\text{codebook}}$, is the codebook loss, which aligns the code vectors in the codebook with the latent representations produced by the encoder. It is defined as:


(4) $$L_{\text{codebook}}={\left\|\text{sg}[z_{e}]-z_{q}\right\|}_{2}^{2},$$

where $z_{e}$ is the latent representation output by the encoder, and $z_{q}$ is its quantized counterpart obtained by mapping $z_{e}$ to the nearest code vector in the codebook. The stop-gradient operator sg[·] ensures that gradients flow only through the codebook, preventing the encoder from directly influencing the code vectors.

The third term, $L_{\text{commit}}$, is the commitment loss, which ensures that the encoder output remains close to the selected code vector to avoid instability during training. It is defined as:


(5) $$L_{\text{commit}}={\left\|z_{e}-\text{sg}[z_{q}]\right\|}_{2}^{2}.$$

The hyperparameter β controls the trade-off between maintaining the encoder’s representational flexibility and stabilizing codebook updates. For most applications, β is typically set to 0.25. By jointly minimizing these losses, the VQ-VAE learns a set of discrete code vectors that can be used as a vocabulary for protein fragment features.

Training VQ-VAE presents inherent difficulties, including codebook collapse and training instability (Łańcucki *et al.* 2020). To avoid these problems, we introduced an Attention-based encoder based on multi-head attention mechanisms. This architecture enables the encoder to capture nuanced, context-aware relationships within protein fragment features, improving the informativeness and stability of the encoding process.

### 2.3 Local structural pattern representation

A critical component in building this vocabulary lies in representing local structural patterns with fixed-dimension feature vectors. To achieve this, we integrated diverse features derived from both protein sequences and structures, including amino acid composition, evolutionary conservation profiles, and three-dimensional spatial geometry. This multifaceted approach ensures a biologically grounded representation of structural patterns, balancing granularity with functional relevance.

**Amino acid composition features**: a 20-dimensional vector, describing the types and proportions of amino acids in a local structural fragment. For a given protein structural fragment containing *n* amino acids, its amino acid composition is expressed as a vector $v=[e_{1},e_{2},\ldots ,e_{20}]$, where each element $e_{i}$ represents the percentage of the *i*-th standard amino acid in the fragment.

**Evolutionary conservation features**: two types of evolutionary conservation profiles, the Position-Specific Scoring Matrix (PSSM) and the Hidden Markov Model (HMM) profile from pairwise alignments, generated using PSI-BLAST (Altschul *et al.* 1997) [search the NCBI (Sayers *et al.* 2009) non-redundant database with five iterations and an *e*-value threshold of 0.001] and HHblits (Remmert *et al.* 2012) [search the UniRef30 database (Suzek *et al.* 2015) with default parameters], respectively. Each row in the PSSM corresponds to a residue and contains 20 elements representing substitution scores for different amino acids. These scores are normalized using a sigmoid function for consistent scaling. We extract features from HMM profiles following iNucRes-ASSH (Zhang *et al.* 2024). The PSSM and HMM profiles for a protein fragment are expressed as matrices of size n×20, where *n* is the number of amino acids in the fragment. Column-wise averaging of each matrix produces two 20-dimensional vectors. The final evolutionary conservation feature for the fragment is obtained by concatenating these two vectors into a single representation.

**Spatial geometric information features**: a 14-dimensional vector, primarily comprising information on secondary structure types and torsion angles between residues. Specifically, an 8-dimensional one-hot vector is used for each fragment to encode the eight types of protein secondary structures. Additionally, six residue-specific features are extracted from the DSSP tool, including solvent-accessible surface area and torsion angle characteristics. To generate fragment-level features, the average value of each feature across all residues within the fragment is computed, providing a global representation of the spatial geometric properties of the fragment.

**Figure 2.**
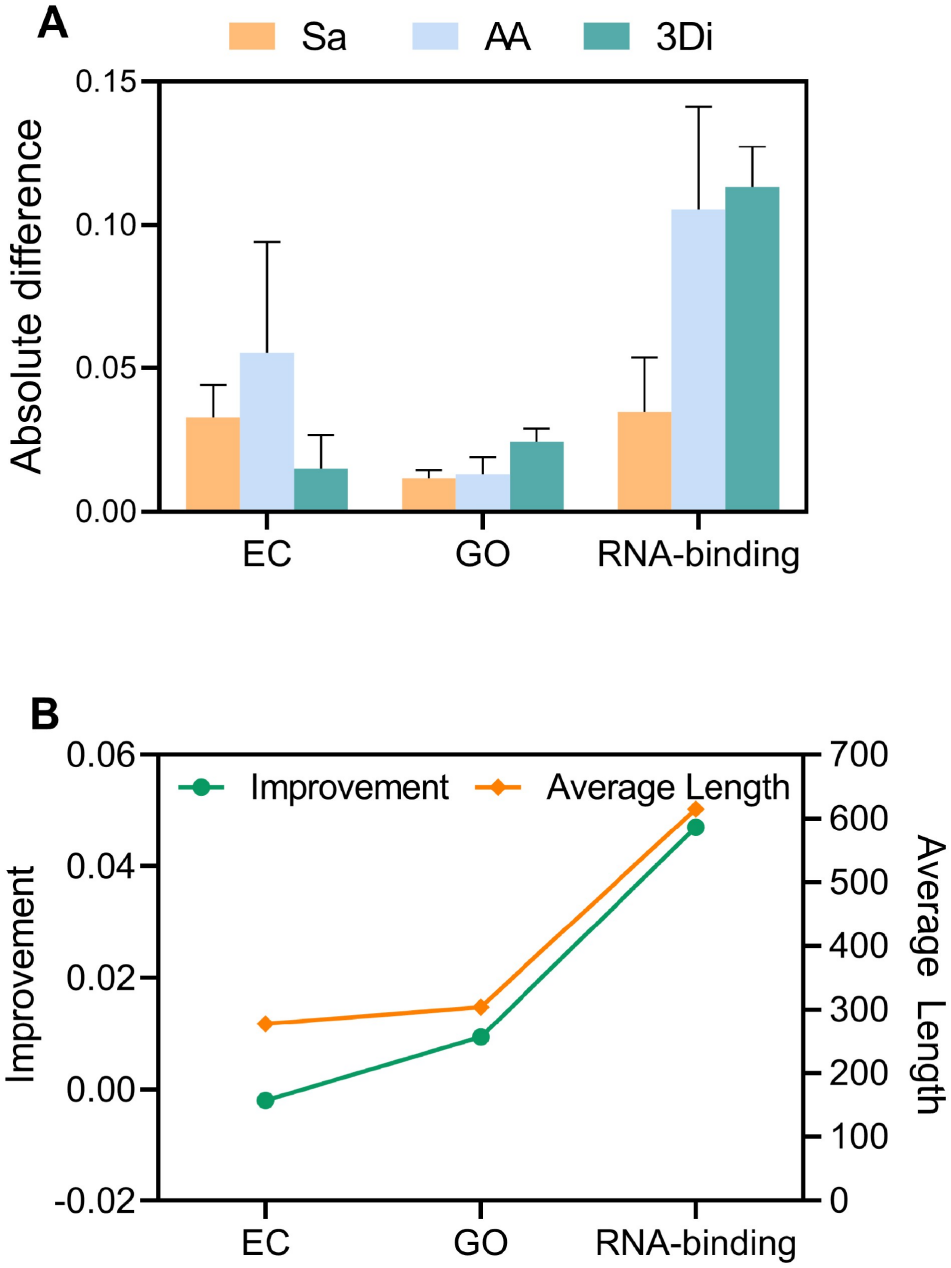
Results on the influence of protein length on protein modeling. (A) Absolute performance differences in downstream tasks (EC, GO, RNA-binding) when using representations from language models pre-trained with 512-token versus 1024-token truncation lengths, evaluated on test-set proteins exceeding 512 residues. (B) The relationship between SCG’s improvement over Sa on large proteins and the average protein length of the task test dataset (Pearson’s *r *= 0.9884).

By concatenating the above three kinds of features, we get a 74-dimensional feature for each fragment as the input of VQ-VAE. Such features comprehensively capture the key attributes of fragments at both the sequence and structural levels, providing a rich semantic representation for vocabulary construction (Fig. 2, available as supplementary data at *Bioinformatics* online).

### 2.4 Serialization of protein

For a given protein, we employ the aforementioned protein structure segmentation method to discretize it into multiple structure fragments. Subsequently, utilizing a pre-trained VQ-VAE model and the derived coarse-grained vocabulary, each structure fragment is mapped to a specific token. Finally, the corresponding tokens of these structure fragments are sequentially assembled into a protein sentence based on the positions of these fragments within the protein sequence.

### 2.5 Datasets

The study employs two distinct data categories to support model development and evaluation:

1. **Pretraining Dataset** 
  We obtained 206,938 protein structures from the Protein Data Bank (PDB, downloaded in March 2024, the most recent version available at that time) (Berman *et al.* 2000). To ensure data independence and applicability, we performed chain-splitting processing on these structures, decomposing multi-chain proteins into single-chain forms. Subsequently, we applied CD-HIT (Fu *et al.* 2012) with parameters set to “-c 0.98 -aS 0.95” to remove redundant sequences from all single chains, resulting in 104,774 protein single chains, which serve as the pre-training benchmark dataset.
2. **Datasets of Downstream Tasks** 
  To evaluate the proposed method, we employ three downstream tasks: Gene Ontology (GO) term prediction, Enzyme Commission (EC) classification (Gligorijević *et al.* 2021), and RNA-binding identification. The detailed statistics of these datasets are provided in Table 1. The GO term prediction task aims to classify proteins into functional categories based on the Gene Ontology, which is organized into three ontologies: Molecular Function (MF), Biological Process (BP), and Cellular Component (CC) (Ashburner *et al.* 2000). The EC classification task focuses on predicting the catalytic functions of enzymes, where EC numbers describe the biochemical reactions catalyzed by enzymes. We adopted the data split in Zhang *et al.* (2023) for the GO and EC evaluation, excluding anomalous protein structures, such as those consisting solely of carbon α atoms.

**Table 1.** Dataset statistics for downstream tasks.

	Train	Validation	Test
Gene ontology	29 896	3179	3233
Enzyme commission	15 396	1711	1891
RNA-binding	6365	1084	1366

We constructed the RNA-binding dataset by extracting all RNA-binding protein complexes from the Protein Data Bank. From these, we identified approximately 20,000 protein chains that are known to bind RNA as positive samples. For negative samples, we selected non-RNA-binding proteins from the PDB, excluding any chains annotated as RNA-binding in UniProt (Uniprot Consortium 2022). We used CD-HIT to reduce sequence redundancy, applying a sequence identity cutoff of 0.95 for the positive samples and a more stringent cutoff of 0.75 for the negative samples to retain sufficient data diversity. The final dataset consists of a balanced number of positive and negative samples. This task is formulated as a binary classification problem, where the objective is to determine whether a given protein can bind RNA. A performance comparison under stricter redundancy control (40% sequence identity threshold) between train and test datasets is provided in Table 5, available as supplementary data at *Bioinformatics* online.

### 2.6 Experimental setup

To evaluate the universality and effectiveness of the SCG protein language, we benchmarked it using two distinct language modeling architectures: Doc2Vec (Le and Mikolov 2014), a lightweight embedding model suitable for efficient sequence representation, and BERT (6-layer, refer to Table 1, available as supplementary data at *Bioinformatics* online), a Transformer-based architecture widely adopted for deep contextual learning [exemplified by the ESM framework (Rives *et al.* 2021)]. This dual-model approach ensures robustness across computational scales, validating whether the proposed coarse-grained language generalizes to both parameter-efficient and resource-intensive architectures. We compared the SCG language against existing protein languages (e.g., residue-level and hybrid sequence-structure languages) across the aforementioned three downstream tasks critical to functional analysis.

**Related works**. We compare the SCG protein language with three fine-grained protein languages: (i) the amino acid sequence-based protein language (AA) widely used in PLMs, such as ESM2, (ii) the structure-aware language (Sa) used in SaProt, and (iii) the structural conformation language based on the 3Di vocabulary proposed in Foldseek (3Di). Additionally, the coarse-grained protein language based on the BPE Tokenizer from PETA (Tan *et al.* 2024) (BPE) is also used as one of the comparative baselines.

**PLM training**. Throughout the experiments, we use the same pretraining dataset with 104,774 protein structures and maintain consistent training configurations for all language models to ensure a fair comparison. We train PLMs based on different protein languages and extract embeddings from these PLMs for downstream task datasets to evaluate their effectiveness without performing fine-tuning. For downstream task prediction, we use an MLP as the classifier. The training details and hyperparameters are provided in Table 2, available as supplementary data at *Bioinformatics* online, and the representative learning curves are shown in Fig. 1, available as supplementary data at *Bioinformatics* online.

## 3 Results

### 3.1 Benchmark various languages

We used the Fmax score (see Supplementary Materials, available as supplementary data at *Bioinformatics* online) to measure the prediction of GO and EC, and the AUC-ROC score to measure the prediction of RNA binding. For an objective evaluation, we conducted three independent experimental trials for each model and reported the mean and standard deviation of the results across these trials. The results of different languages are listed in Table 2.

**Table 2.** Performance comparison across different protein language representations on different language models.^a^

LM	Method	Type	GO-BP	GO-MF	GO-CC	EC	RNA-binding
BERT-based	AA	Fine	0.372±0.0006***	0.392±0.0009***	0.410±0.0021	0.562± 0.0026***	0.829±0.0021**
	3Di	Fine	0.405±0.0015***	**0.501** ± 0.0078	0.391±0.0034**	**0.730** ± 0.0044**	0.814±0.0042***
	Sa	Fine	0.412 ± 0.0025***	0.488±0.0027**	**0.418** ± 0.0024*	0.717 ±0.0038*	0.850 ±0.0017*
	BPE	Coarse	0.362±0.0062***	0.381±0.0052***	0.403±0.0011*	0.567±0.0025***	0.827±0.0031**
	SCG	Coarse	**0.425** ± 0.0009	0.500 ±0.0002	0.411 ±0.0023	0.705±0.0047	**0.863** ± 0.0027
Doc2Vec	AA	Fine	0.121±0.0028***	0.110±0.0043***	0.258±0.0154***	0.379±0.0038***	0.792±0.0083
	3Di	Fine	0.110±0.0125***	0.101±0.016***	0.269±0.0188**	0.427±0.0012***	0.726±0.0014***
	Sa	Fine	0.327 ±0.0020**	0.335 ±0.0060***	0.347 ±0.0038	0.537 ±0.0023*	0.800 ±0.0118
	BPE	Coarse	0.121±0.0125***	0.095±0.016***	0.250±0.0092***	0.377±0.0015***	0.790±0.0046*
	SCG	Coarse	0.340±0.0009	0.380±0.0027	0.351±0.0048	0.551±0.0017	0.804±0.0039

a Bold numbers indicate the best performance in each category, and underlined values denote the second-best. The “Type” column denotes representation granularity: fine for fine-grained and coarse for coarse-grained representations. *Note*: AA is used in ESM series language models; 3Di is driven from Foldseek; Sa is used in SaProt; and BPE is a classic NLP modeling method. Statistical significance was assessed using Welch’s *t*-tests. Significance levels are indicated as follows:

* P<0.05 ,

** P<0.01 ,

*** P<0.001 , blank indicates not significant.

Under the Bert-based model, SCG achieves leading performance in biological process prediction and RNA-binding tasks and achieves second-best performance in GO-MF and GO-CC tasks. Its third-place ranking in EC prediction suggests that shorter sequences in this dataset may limit SCG’s structural abstraction benefits. When applied to the Doc2Vec model, SCG exhibits more consistent advantages, outperforming alternative representations across all tasks. This suggests that the proposed coarse-grained encoding aligns particularly well with statistical learning frameworks, where structural abstraction may help mitigate noise in sequence-based representations. SCG language achieves stable performance regardless of the underlying model, reinforcing its generalizability as a protein language representation.

While BPE (Sennrich *et al.* 2016)—a widely used NLP tokenization method—fails to surpass residue-level (AA) representations, SCG’s integration of secondary structural priors yields superior semantic expressiveness. This highlights the necessity of embedding biological knowledge into protein language design.

Structure-based languages (e.g., 3Di) excel in tasks like GO-MF and EC prediction under BERT. However, hybrid approaches like SaProt (Sa) show no consistent advantage, suggesting task-specific dependencies on sequence-structure integration. SCG’s competitive performance across all tasks—despite minimal structural detail—emphasizes the critical role of conserved local patterns in functional prediction. Further experimental results on protein structural family classification are provided in Table 3, available as supplementary data at *Bioinformatics* online, further validating the state-of-the-art performance of SCG.

### 3.2 The influence of length on protein modeling

By leveraging secondary structure-based segmentation, SCG sentences are significantly shorter than fine-grained protein language sequences. Statistical analysis of the pre-training dataset revealed that fine-grained sequences are, on average, 7.28 times longer than SCG sentences. To standardize comparisons, we truncated SCG sequences exceeding 512 tokens, while retaining the original maximum length of 1024 for other languages (consistent with original protocols). Under this configuration, only 5 SCG sequences required truncation, compared to 1,367 truncated sequences in fine-grained languages.

To assess the impact of sequence truncation, we evaluated three fine-grained protein languages (Sa, AA, and 3Di) on downstream tasks using two configurations: a truncated input length of 512 tokens (applied to sequences exceeding this limit) and the original maximum length of 1024 tokens. The absolute difference in performance between these configurations quantified how truncation compromises semantic learning. All fine-grained languages showed measurable truncation effects (absolute difference > 0, Fig. 2A). These findings demonstrate that truncation compromises the model’s ability to capture semantic and contextual information. In contrast, SCG sentences are rarely subject to truncation because their lengths are substantially shorter than those of raw amino acid sequences. As a result, SCG representations experience minimal truncation effects, which helps preserve structural and functional context in downstream tasks.

**Figure 3.**
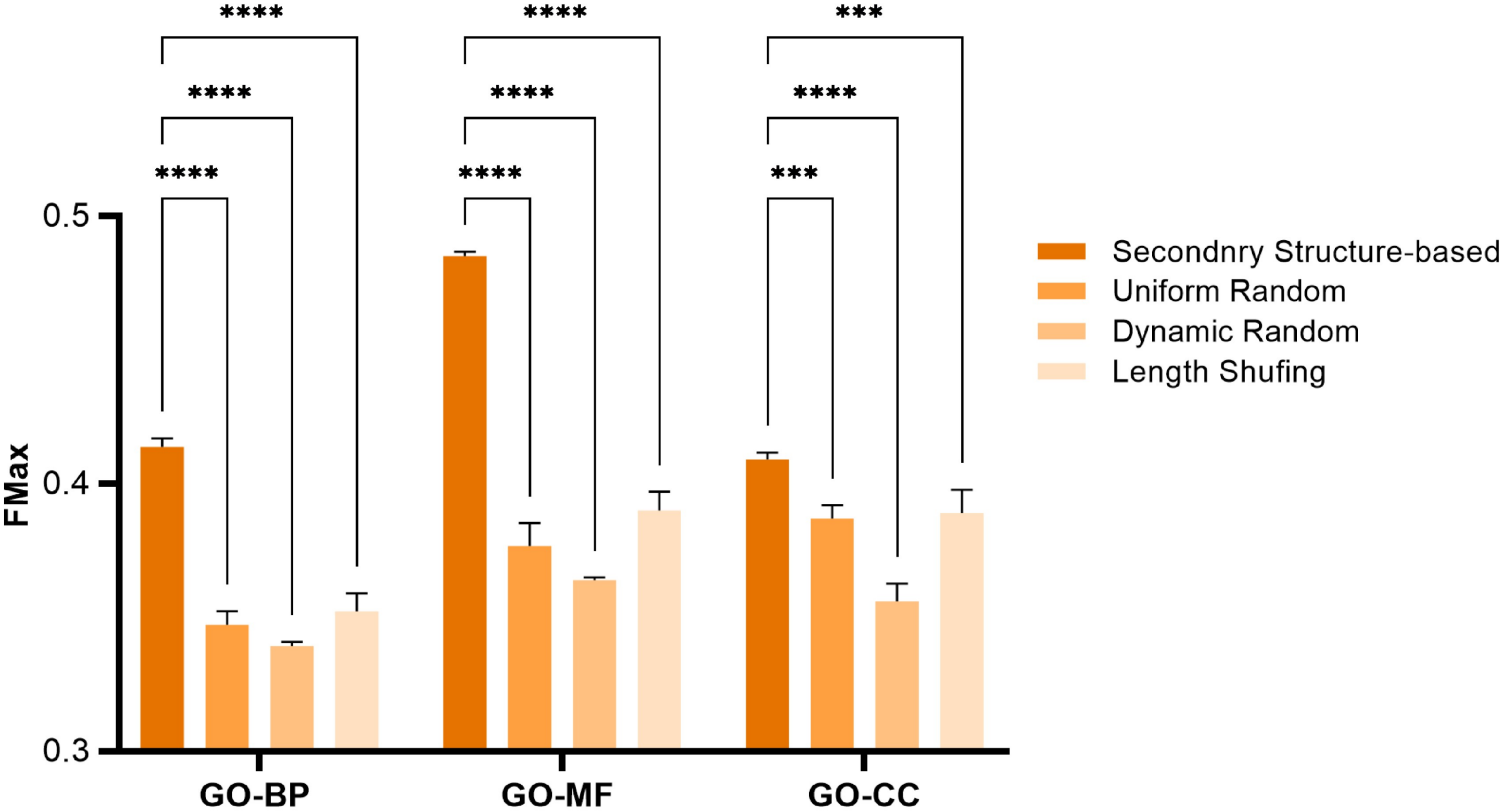
Evaluation of different segmentation strategies for SCG. SCG uses secondary structure-based segmentation, and other methods are designed for comparison. Uniform Random denotes Uniform Random Fragment Length Segmentation, similar to Dynamic Random. Length Shuffling denotes Secondary Structure Fragment Length Shuffling Segmentation. The symbols “***” and “****” denote statistical significance levels: specifically, “***” indicates a p-value < 0.001, and “****” indicates a p-value < 0.0001.

Theoretical considerations suggest that SCG’s structure-aware coarse-grained representation offers distinct advantages for modeling long proteins. To empirically validate this hypothesis, we further evaluated SCG’s performance across proteins of varying lengths. Following the protocol (Hu *et al.* 2024), we categorized the proteins in the test set into long and short groups using the dataset’s average sequence length as a threshold (Table 3). We specifically compared SCG against the state-of-the-art fine-grained language Sa by analyzing performance disparities along with average protein length. As illustrated in Fig. 2B, SCG’s improvement over Sa correlates strongly with protein length: longer sequences consistently yielded greater performance gains, empirically suggesting SCG’s representational advantages for long proteins. This trend is further verified in Fig. 3, available as supplementary data at *Bioinformatics* online, which plots the relative performance gain of SCG over Sa in short proteins (less than 200 residues) and long proteins (more than 500 residues). Although Sa outperforms SCG in GO-CC and EC tasks in terms of absolute performance (Table 4, available as supplementary data at *Bioinformatics* online), the performance gain of SCG on long proteins is consistently greater than that on short proteins (Fig. 3, available as supplementary data at *Bioinformatics* online). This overall trend further confirms that SCG benefits more from long protein sequences.

**Table 3.** Distribution of small and large proteins, along with their average lengths across three downstream tasks.

Task	Short	Long	Average length
GO	1802	1431	304
EC	1068	823	278
RNA-binding	897	469	615

### 3.3 Exploration of segmentation schemes

To validate the rationale behind protein secondary structure-based segmentation, we compared it with three distinct segmentation strategies:

- *Uniform Random Fragment Segmentation*: For each protein sequence, a fragment length is randomly selected from the range [3, 60], and the sequence is evenly divided into fragments of this length. Except for the last fragment, all other fragments have the same length.
- *Dynamic Random Fragment Segmentation*: At each segmentation step, a fragment length is randomly selected from the range [3, 60], and the protein sequence is divided accordingly. This process is repeated until the entire sequence is fully segmented.
- *Secondary Structure Fragment Shuffling Segmentation*: We collect the fragment lengths derived from secondary structure-based segmentation and randomize the order of these lengths. The protein sequence is segmented according to the shuffled fragment length set.

We evaluated different segmentation schemes via GO prediction. For each segmentation scheme, we reprocessed the dataset, constructed the corresponding vocabulary, and trained the independent language model from scratch to ensure fair comparability across methods. The detailed training settings are provided in the Supplementary Material, available as supplementary data at *Bioinformatics* online, Training Details Section (Vocabulary Training). The results are shown in Fig. 3, demonstrating the effectiveness of secondary structure-based segmentation compared to three alternative schemes. Uniform Random divides sequences into fixed-length fragments, while Dynamic Random uses variable-length fragments at each step, both leading to significant performance degradation due to the disruption of biologically meaningful patterns. Length Shuffling, which retains the same number and lengths of fragments as secondary structure-based segmentation but alters their order, also underperforms. This indicates that not only the fragment lengths but also their sequence-specific arrangement are critical for capturing functional protein representations. These results highlight that domain-specific segmentation, guided by secondary structure, is essential for preserving biologically relevant features and achieving robust performance in protein language models.

### 3.4 Study on vocabulary

**Figure 4.**
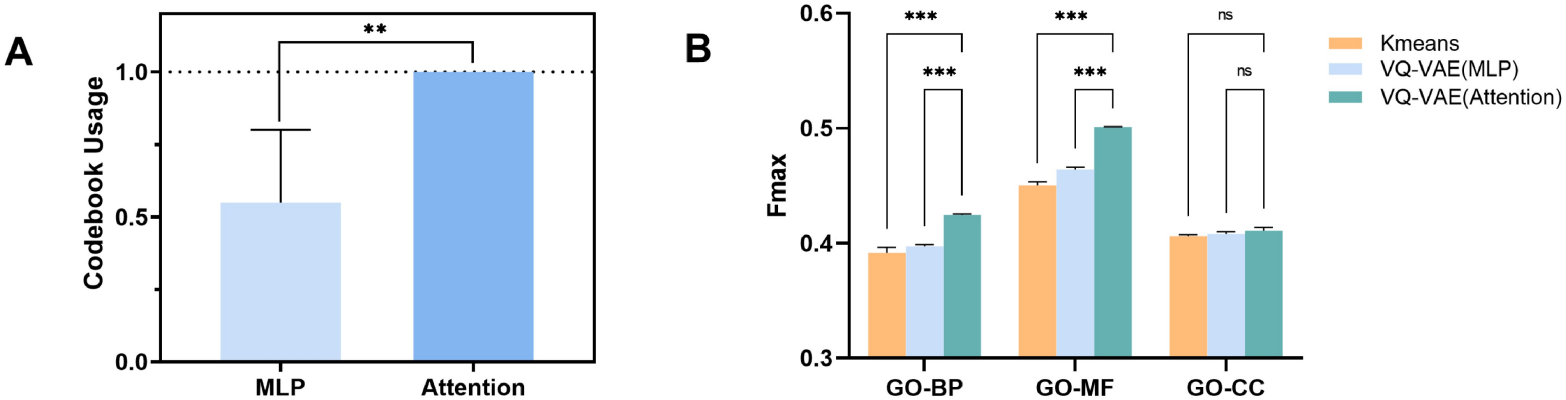
Results of ablation studies on vocabulary construction. (A) Comparison of codebook usage between VQ-VAE models using a feedforward MLP encoder and a multi-head attention encoder. The attention-based variant achieves full codebook utilization, indicating improved stability and efficiency in vector quantization. (B) Performance evaluation (Fmax) on GO tasks using vocabularies trained by different methods, including k-means, VQ-VAE (MLP), and VQ-VAE (Attention). The symbols “**” and “***” denote statistical significance levels: specifically, “**” indicates a p-value < 0.01, and “***” indicates a p-value < 0.001.

To address training instability and codebook collapse in VQ-VAEs, we integrated a multi-head attention mechanism into the encoder. To validate this design, we compared two VQ-VAE variants: one using a feedforward neural network (MLP) encoder and another with the attention-based encoder. Experiments confirmed that the attention mechanism substantially improves codebook utilization (i.e., more code vectors are actively engaged during training, Fig. 4A), which directly enhances vocabulary construction efficacy.

While traditional clustering methods like k-means could theoretically generate vocabularies, we evaluated their performance against VQ-VAE by training BERT language models on vocabularies derived from both approaches. Results from GO prediction tasks demonstrated that VQ-VAE consistently outperformed k-means (Fig. 4B). This advantage likely stems from VQ-VAE’s ability to learn latent features during vocabulary construction, whereas k-means relies solely on static input features, limiting its capacity to capture nuanced structural patterns.

To evaluate the impact of vocabulary size on the SCG method’s performance, we conducted an ablation study across GO prediction tasks (results in Table 7, available as supplementary data at *Bioinformatics* online). Balancing the trade-offs between vocabulary expressiveness and computational efficiency, 1024 tokens emerge as the optimal choice for protein language modeling. This size ensures robust performance across all GO tasks without compromising scalability.

## 4 Conclusion and discussion

This study introduces a structure-aware coarse-grained (SCG) protein language that integrates secondary structure segmentation with vector quantization techniques to redefine protein representation. Our framework partitions proteins into biologically meaningful structural fragments, reducing sequence length while preserving functional semantics. By constructing a compact vocabulary of these fragments, the SCG language mitigates semantic truncation in large proteins during language model processing. Empirical evaluations and ablation studies confirm that the SCG language outperforms traditional amino acid sequences and state-of-the-art protein languages in capturing molecular-level semantics, particularly for long proteins.

While numerous protein modeling approaches have been developed in recent years, they differ substantially in paradigm and research objectives. Large-scale pretrained sequence models, such as ESM-2 (Lin *et al.* 2023), ESM-C (https://evolutionaryscale.ai/blog/esm-cambrian), and ProtT5 (Elnaggar *et al.* 2022), have achieved remarkable success by leveraging massive protein sequence corpora to learn generalizable representations. Other approaches integrate explicit structural information, including graph-based architectures such as GearNet (Zhang *et al.* 2023) and structure-aware models like DeepFRI (Gligorijević *et al.* 2021) and ProstT5 (Heinzinger *et al.* 2024), which jointly utilize sequence and structural modalities to enhance protein function or fold prediction.

SCG, in contrast, focuses on the linguistic design of protein representations—specifically, how granularity and structural awareness influence the expressiveness and efficiency of protein language models. Rather than relying on large-scale data or graph-based structural modeling, SCG explores a complementary perspective centered on structure-guided tokenization. Therefore, direct comparisons with the aforementioned models would not provide meaningful insights from the language design standpoint pursued in this work. Nevertheless, the SCG representation could potentially be combined with these approaches in future work to further improve modeling efficiency and performance.

Despite the demonstrated advantages and conceptual complementarity of the SCG framework, several limitations remain to be addressed: (i) The vocabulary construction is based on protein fragment features, which rely on manually designed features and require additional parsing of materials such as DSSP and evolutionary conservation profiles. This preprocessing step can be time-consuming and also requires some memory; the resource consumption is provided in Table 6, available as supplementary data at *Bioinformatics* online; (ii) Due to computational constraints, we only validated the effectiveness of the proposed language with lightweight models on a small pretraining dataset. The performance of SCG language with deeper models and large-scale data is worthy of further investigation; (iii) Handling proteins with missing or low-quality structural data represents a potential challenge for SCG, as well as a common issue for structure-based modeling approaches. For proteins lacking experimentally determined structures or exhibiting low-quality structural information, a potential solution is to predict or reconstruct their three-dimensional conformations using reliable computational methods, such as AlphaFold3 (Abramson *et al.* 2024).

Beyond language models, the SCG modeling framework could enhance computational efficiency in graph-based protein modeling, where processing large proteins remains resource-intensive. Extending this coarse-grained paradigm to graph neural networks may unlock new opportunities for scalable, structure-aware protein analysis.

## Supplementary Material

btaf654_Supplementary_Data

## Data Availability

All the datasets used in this study and the source code of the proposed method are available at GitHub (https://github.com/bug-0x3f/coarse-grained-protein-language) and Zenodo (DOI: 10.5281/zenodo.17674298).
