# Supplementary material for: Molecular-level protein semantic learning via structure-aware coarse-grained language modeling
Source: Bioinformatics. 2025 Dec 6;42(1):btaf654. doi: 10.1093/bioinformatics/btaf654 (PMC12758601; doi:10.1093/bioinformatics/btaf654)
Supplement: btaf654_Supplementary_Data [file btaf654_supplementary_data.pdf]

Molecular-Level Protein Semantic Learning via Structure-Aware Coarse-Grained Language Modeling

Jun Zhang*, Xueer Weng, Tiantian Zhu, Yumeng Liu and Zexuan Zhu*

1 More Method and Training Details

1.1 Vocabulary Training

Following the methodology outlined in our study, we employ a VQ-VAE framework to construct our structural vocabulary through self-supervised reconstruction of approximately 4 million protein fragments from the pretraining corpus. The model architecture consists of an encoder with an input dimension of 74, a hidden dimension of 240, and an intermediate dimension of 240. The vector quantization layer utilizes a codebook of size 1024, with each embedding vector having a dimension of 10. The model is optimized using AdamW with decay parameters ($\beta_1 = 0.9$, $\beta_2 = 0.99$) and a learning rate of 1×10^{-3}, trained for 20 epochs with a batch size of 256. The commitment loss coefficient β in the equation is set to 0.25, consistent with the original VQ-VAE formulation, ensuring an effective balance between codebook utilization and feature reconstruction. The encoder of the VQ-VAE is implemented as a transformer encoder block, which captures hierarchical structural patterns in protein fragments through self-attention mechanisms.

In the main text, we compare the downstream performance of vocabularies constructed using k-means and VQ-VAE. For k-means clustering, we used the k-means++ initialization method from the scikit-learn library, with all other parameters set to their default values. To ensure a fair comparison, both methods were applied to the same training set to construct vocabularies.

1.2 Language Models Training

The details of the training setting are as follows:

- For Doc2Vec, we construct a corpus from the pretraining dataset. The model was configured with a vector size of 600, a minimum word count of 2, and trained for 150 epochs. The choice of 150 epochs was empirically determined through preliminary exploratory experiments, where we trained models with varying epoch numbers and assessed the quality of their learned embeddings on downstream tasks. We set the initial learning rate (alpha) to 0.025, with a minimum learning rate (min_alpha) of 0.0001, and utilized 256 worker threads for parallel processing.

- The BERT-based model used in this study follows the same architecture as the one employed in ESM2[Lin et al., 2023], though we refer to it as the BERT-based model to avoid confusion. The model configuration consists of 6 layers, 768 hidden units, 6 attention heads, and a feedforward layer dimension of 3072. We trained the model for 50 epochs using the AdamW optimizer with $\beta_1 = 0.9$, $\beta_2 = 0.99$, and a learning rate of 0.0001. The batch size was set to 256, distributed across 4 A6000 GPUs, with a local batch size of 64. To enhance computational efficiency and reduce memory usage, we utilized mixed-precision training. For sequence length truncation, we limited our method to a maximum of 512 tokens, while other models were truncated at 1024 tokens. During training, we employed a masked language modeling approach, where 15% of the input tokens were randomly selected for masking, following the same masking strategy as the original BERT model [Devlin et al., 2019].

The analysis of model size reveals that increasing the number of layers improves performance. As shown in **Table S1**, the 6-layer model outperforms the 3-layer model. The 12-layer model exhibits marginal improvements over the 6-layer model, likely due to the relatively small size of the pre-training dataset. Balancing performance capabilities with computational demands, thus we selected a 6-layer architecture to benchmark across diverse protein languages.

Table S1: Impact of model size on language model training performance.
Bold values indicate the best performance, while underlined values represent the second-best performance.

Model Size	GO-BP	GO-MF	GO-CC
3-layer	0.423±0.0011	0.481±0.0015	0.379±0.0119
6-layer	0.425±0.0009	**0.500**±0.0002	0.411±0.0023
12-layer	**0.426**±0.0054	0.496±0.0014	**0.412**±0.0065

1.3 Downstream Task Details

1.3.1 Downstream Task Training Details

For downstream task prediction, we average the hidden states of the language model to obtain protein representations and use a three-layer MLP as the classifier. For the EC and GO tasks, the hidden layer size of the classifier is set to match the output layer dimension, which corresponds to the number of labels. The Binary Cross-Entropy Loss is adopted for all tasks. Further hyperparameter configurations are provided in **Table S2**. During training, we employ an early stopping mechanism with a patience of 10 epochs. Within the early stopping window, we save the top 5 models based on their performance on the validation set. For the final evaluation on the test set, we compute the average performance of these 5 models to ensure robustness and reduce variance in the results. To illustrate the training dynamics and model convergence, we additionally provide the learning curves of the GO-CC task, showing both the loss and Fmax metrics over training epochs (**Figure S1**).

Table S2: Hyperparameter configurations of different prediction tasks. The batch size reported refers to the global batch size. All the hyperparameters are chosen by the performance on the validation set.

Hyperparameter	GO-BP	GO-MF	GO-CC	EC	RNA-binding
layer	3	3	3	3	3
MLP hidden size	1943	489	320	538	512
batch size	2	2	2	2	32
optimizer	Adam	Adam	Adam	Adam	Adam
learning rate	1e-4	1e-4	1e-4	1e-4	5e-4
epoch	200	200	200	200	200

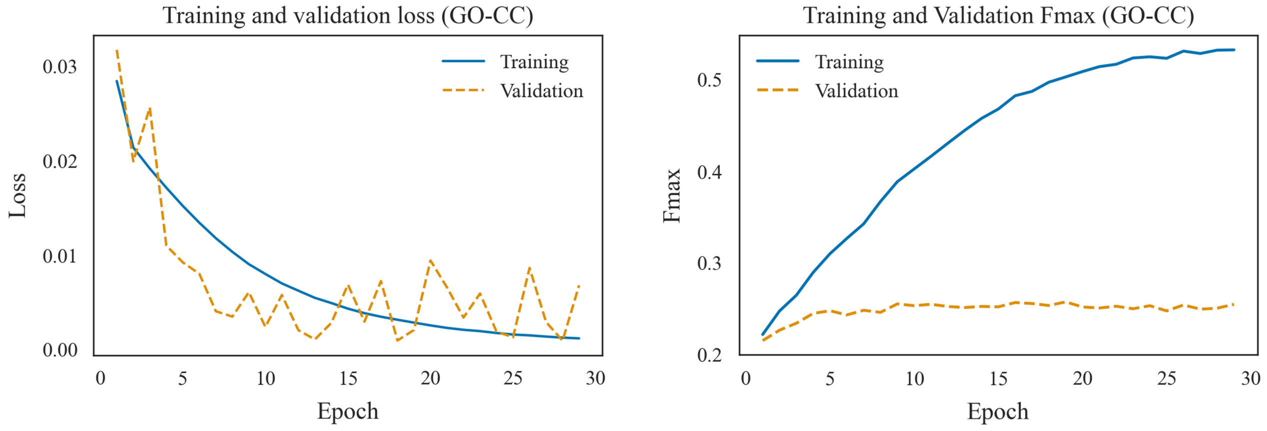

Figure S1: Training and validation curves on the GO-CC task.

1.3.2 Fmax Score

To evaluate the prediction performance for GO and EC annotations, we used the Fmax score. This metric corresponds to the maximum F-score obtained by varying the decision threshold τ over the interval $[0, 1]$.

Let n denote the total number of proteins, and $m(\tau)$ denote the number of proteins with at least one predicted label above threshold τ, i.e., $|p_i(\tau)| > 0$. For each protein i, let $p_i(\tau)$ be the set of predicted labels above threshold τ, and t_i be the set of true labels. The average precision and recall over all proteins at threshold τ are defined as:

$$\mathrm{pr}(\tau) = \frac{1}{m(\tau)} \sum_i \mathrm{pr}_i(\tau), \tag{1}$$

$$\mathrm{rc}(\tau) = \frac{1}{n} \sum_i \mathrm{rc}_i(\tau), \tag{2}$$

where per-protein precision and recall are computed as

$$\mathrm{pr}_i(\tau) = \frac{|p_i(\tau) \cap t_i|}{|p_i(\tau)|}, \quad \mathrm{rc}_i(\tau) = \frac{|p_i(\tau) \cap t_i|}{|t_i|}. \tag{3}$$

The F-score at threshold τ is then given by

$$f(\tau) = \frac{2 \cdot \mathrm{pr}(\tau) \cdot \mathrm{rc}(\tau)}{\mathrm{pr}(\tau) + \mathrm{rc}(\tau)}. \tag{4}$$

Finally, the Fmax score is defined as the maximum F-score across all thresholds:

$$F_{\max} = \max_{\tau} f(\tau). \tag{5}$$

1.4 More Details about Segmentation

In the SCG segmentation process, each protein chain is decomposed into structural fragments based on secondary structure boundaries and residue continuity. Fragments shorter than three residues are filtered out to avoid unstable or uninformative patterns. However, to ensure that every protein can still obtain a valid SCG representation, the filtered short residues—as well as excessively long uninterrupted residue regions (longer than 60 residues)—are regrouped and treated as independent fragments. This strategy ensures that every protein, regardless of its structural completeness or irregularity, can be encoded into an SCG sentence.

Notably, there is no strict lower bound on protein sequence length in SCG. Nevertheless, extremely short sequences are generally not recommended for SCG representation, as the absence of consistent secondary structure motifs may compromise the reliability and robustness of the resulting embeddings.

2 Further Experimental Analyses

2.1 Fragment Feature Ablation Study

As described in the main text, we extract three kinds of features from local structure patterns for learning SCG vocabulary. In this subsection, we conducted an ablation analysis by removing one feature at a time and comparing the GO term prediction performance to evaluate the importance of each feature. The two evolutionary conservation profiles were studied separately. As shown in Supplementary **Figure S2**, structural and evolutionary features play crucial roles in improving protein function prediction, especially for GO-CC. Structural and amino acid composition features are significant for capturing detailed functional information for GO-BP and GO-CC. PSSM feature has a smaller impact on overall performance but still contributes valuable sequence-level information. The result confirms that the SCG vocabulary benefits from a multi-faceted feature set that captures both the sequence and structural properties of proteins.

2.2 Protein Structural Family Classification Experiment

To directly evaluate whether the proposed SCG representation captures protein structural information more effectively than sequence-only baselines, we conducted a protein structural family classification experiment.

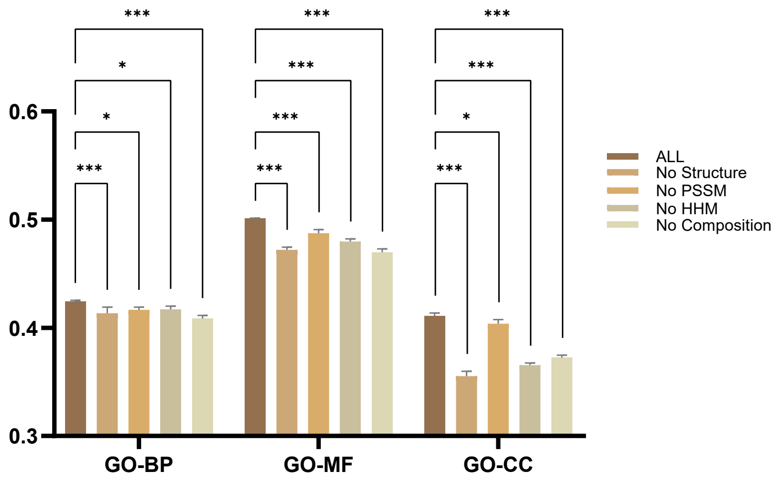

Figure S2: Results on fragment feature ablation

2.2.1 Dataset Construction

Protein chains belonging to three representative structural families—Alpha, Beta, and Alpha-Beta—were downloaded directly from the Protein Data Bank (PDB, accessed on August 3, 2025). In total, 39,040 protein chains were collected, of which 22,485 overlapped with the pretraining dataset and 16,555 were newly obtained sequences. To eliminate redundancy between pretraining and evaluation data, CD-HIT-2D was applied between the two subsets using parameters "-c 0.4 -n 2", resulting in 973 non-redundant protein chains as the final independent test set. Proteins within the pretraining subset were randomly split into a training set (20,236 chains) and a validation set (2,249 chains) with a 9:1 ratio.

2.2.2 Experiment Setup

Proteins are tokenized using SCG or baseline schemes (AA, BPE, Sa, 3Di) and fed into the pretrained BERT-based protein language model, with token-level hidden states averaged to produce fixed-length embeddings, which were then used to train an MLP classifier for protein structural family prediction (Alpha, Beta, Alpha-Beta).

The MLP consisted of two fully connected layers with 128 hidden units per layer, using ReLU activation. A dropout rate of 0.2 was applied to prevent overfitting. The output layer employed a softmax function over the three family classes. The model was trained for 100 epochs with a batch size of 32. Optimization was performed using AdamW optimizer with a learning rate of 0.0001. Early stopping was applied with a patience window of 10 epochs, meaning that if the validation performance did not improve for 10 consecutive epochs, training was terminated to prevent overfitting. Cross-entropy loss was used as the objective function.

During training, the three models that achieved the highest validation performance were saved. The final test score was computed as the average of their predictions on the test set, which served as the overall performance metric.

2.2.3 Results and Analysis

The performance of the MLP classifier on the independent test set is summarized in **Table S3**.Accuracy (Acc) is used as the primary evaluation metric. Among all tested representations, SCG embeddings achieve the highest accuracy, outperforming traditional sequence-based representations, including AA and BPE. Compared with other advanced representations, SCG slightly outperforms 3Di and is on par with Sa. These results indicate that SCG effectively captures global secondary structure information of proteins, encoding structural distinctions that are difficult to capture with sequence-only representations. While 3Di and Sa benefit from explicit geometric information, SCG achieves competitive performance using only structure-aware coarse-grained language representations combined with simple structural fragment features, highlighting its efficiency and structural sensitivity. In contrast, the sequence-only AA and BPE representations scored lower, confirming that protein embeddings trained with SCG possess the ability to discriminate among protein structural families.

Table S3: Performance of different methods in the protein structural family classification task.

Method	ACC
SCG	**0.785** ± **0.0022**
Sa	**0.785** ± **0.0073**
AA	0.665 ± 0.0097
3Di	0.777 ± 0.0034
BPE	0.683 ± 0.0084

2.3 More Analysis about the Influence of Length on Protein Modeling

To further evaluate whether the performance of SCG is influenced by protein sequence length, we divided the test sequences of each task into two subsets: short sequences (<200 residues) and long sequences (>500 residues), and analyzed the performance improvement of SCG over Sa.

As shown in **Figure S3**, SCG generally performs better on long sequences than on short ones, with particularly notable improvements observed in the RNA-binding and GO-MF tasks. The absolute performance of SCG and Sa is listed in **Table S4**. Although Sa outperforms SCG in GO-CC and EC tasks in terms of absolute performance, the performance gain of SCG on long proteins is consistently greater than that on short proteins (**Figure S3**). This overall trend further confirms that SCG benefits more from long protein sequences.

This trend suggests that SCG's coarse-grained structure-aware representation is more effective in capturing functional and structural information in longer proteins, thereby mitigating the information dilution problem typically associated with extended sequences. In contrast, for shorter sequences, SCG shows relatively weaker performance but remains broadly comparable to Sa. Overall, these results confirm the length-dependent advantage of SCG — it achieves superior performance on long protein sequences while maintaining competitive results on shorter ones.

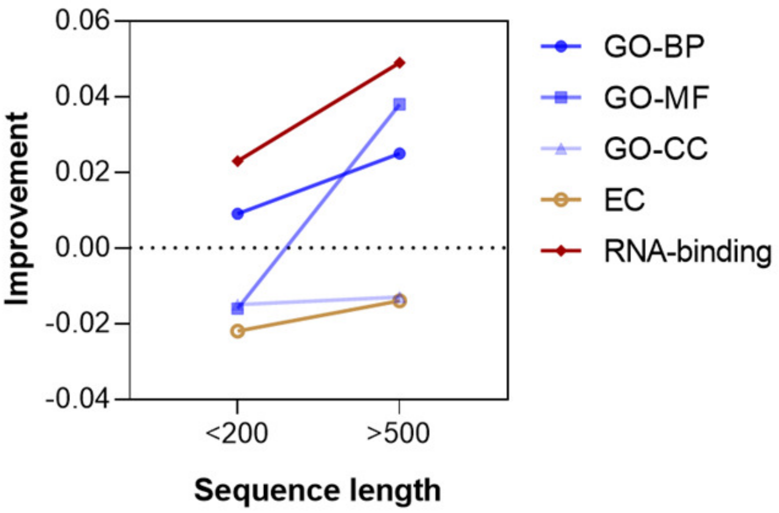

Figure S3: Performance improvement of SCG over Sa across different sequence length subsets.

2.4 RNA Binding Evaluation Under Stricter Redundancy Control

To further assess whether the observed performance improvements on the RNA-binding prediction task were influenced by potential redundancy between the training and test datasets, we conducted an additional experiment under stricter redundancy control. Specifically, we employed CD-HIT-2D to filter out test sequences that shared significant similarity with any training sequence. Due to the tool's lower limit, we used the parameters -c 0.4 -n 2.

After redundancy removal, the test set contained 389 non-redundant sequences. We then re-evaluated all models following the same protocol as in the main experiment. The results (**Table S5**) show that SCG is consistently comparable with Sa and outperforms other baseline approaches across all representation types, and the relative performance rankings remain unchanged.

Table S4: Performance comparison between SCG and Sa on stratified test subsets. Sequences were divided into **long** (>500 residues) and **short** (<200 residues) categories. Metrics are reported as mean ± standard deviation.

Subset	Model	GO-BP	GO-MF	GO-CC	EC	RNA-binding
Long	SCG	**0.413 ± 0.0016**	**0.526 ± 0.0038**	0.401 ± 0.004	0.770 ± 0.0023	**0.843 ± 0.0063**
	SaProt	0.388 ± 0.0028	0.488 ± 0.0071	**0.414 ± 0.005**	**0.784 ± 0.0054**	0.794 ± 0.0079
Short	SCG	**0.436 ± 0.0010**	0.453 ± 0.0035	0.417 ± 0.0022	0.624 ± 0.0056	**0.926 ± 0.0021**
	SaProt	0.427 ± 0.0024	**0.469 ± 0.0014**	**0.432 ± 0.0021**	**0.646 ± 0.0035**	0.903 ± 0.0036
All	SCG	**0.425 ± 0.0009**	**0.500 ± 0.0002**	0.411 ± 0.0023	0.705 ± 0.0047	**0.860 ± 0.0056**
	SaProt	0.412 ± 0.0025	0.488 ± 0.0027	**0.418 ± 0.0024**	**0.721 ± 0.0030**	0.846 ± 0.0035

These results demonstrate that the advantages of our approach in RNA-binding prediction are robust and not attributable to data redundancy between the training and test sets.

Table S5: Performance comparison on the RNA-binding prediction task under stricter redundancy control (40% identity threshold).

**Representation**	**BERT-based**	**Doc2Vec**
AA	0.770 ± 0.0186**	0.766 ± 0.0076
3Di	0.775 ± 0.0145**	0.755 ± 0.0142*
Sa	0.822 ± 0.0095	**0.775 ± 0.0063**
BPE	0.793 ± 0.0049***	0.737 ± 0.0033***
SCG	**0.838** ± 0.0056	**0.775 ± 0.0072**

3 Computational Cost Analysis for Preprocessing and Pretraining

A primary limitation of our method lies in the substantial computational overhead incurred during the preprocessing stage, which is mainly attributable to the calculation of DSSP, PSSM, and HHM features. In this section, we provide a comparative analysis of the preprocessing and training times between our approach and several baseline methods. For the preprocessing evaluation, 100 protein sequences were randomly selected to measure the runtime of each step, whereas the training time was assessed using the complete dataset. All preprocessing experiments were conducted on a Linux server equipped with dual AMD EPYC 7642 processors (2 × 48 cores, 2.30 GHz base frequency, 192 logical cores in total). The GPUs used for pretraining are described in the Language Model Training section of the supplementary material. The results are listed in the table below.

Table S6: Comparison of Preprocessing and Pretraining Efficiency across Different Methods

**Stage**	**Preprocessing**			**Pretraining**	
**Method**	**Time**	**Memory Usage**	**CPU Utilization**	**Time (min)**	**Allocated Memory per GPU**
SCG	432s	40 GB	50.79%	81	8.14 GB
Sa	2s	-	-	98	12.04 GB
AA	-	-	-	129	15.87 GB
3Di	2s	-	-	98	15.95 GB
BPE	-	-	-	215	38.90 GB

Note: "-" indicates that the method requires negligible resources or computation time for the corresponding stage.

Table S7: Analysis of vocabulary size in SCG for GO term prediction. Bold values indicate the best performance, while underlined values represent the second-best performance. Balancing the trade-offs between vocabulary expressiveness and computational efficiency, 1024 emerges as the optimal choice for protein language modeling.

Vocab Size	GO-BP	GO-MF	GO-CC	Average
20	0.367±0.0011	0.441±0.0019	0.390±0.0009	0.399
128	0.405±0.0053	0.470±0.0043	**0.412**±0.0026	0.429
256	0.421±0.0031	0.495±0.0031	0.402±0.0047	0.439
512	0.418±0.0027	0.492±0.0014	0.391±0.0103	0.434
1024	**0.425**±0.0009	0.500±0.0002	0.411±0.0023	**0.445**
2048	0.415±0.0034	0.498±0.0027	0.365±0.0083	0.426
4096	0.418±0.0039	**0.501±0.0015**	0.395±0.0019	0.438
8192	0.423±0.0071	0.496±0.0033	0.365±0.0052	0.428

References

J. Devlin, M.-W. Chang, K. Lee, and K. Toutanova. Bert: Pre-training of deep bidirectional transformers for language understanding, 2019.

Z. Lin, H. Akin, R. Rao, B. Hie, Z. Zhu, W. Lu, N. Smetanin, R. Verkuil, O. Kabeli, Y. Shmueli, A. dos Santos Costa, M. Fazel-Zarandi, T. Sercu, S. Candido, and A. Rives. Evolutionary-scale prediction of atomic-level protein structure with a language model. *Science*, 379(6637):1123–1130, 2023.
